# Tannic acid acts as an agonist of the dopamine D2L receptor, regulates immune responses, and ameliorates experimentally induced colitis in mice

**DOI:** 10.1016/j.bbih.2020.100071

**Published:** 2020-04-30

**Authors:** Masaaki Kawano, Kikue Saika, Rie Takagi, Masanori Matsui, Sho Matsushita

**Affiliations:** a Department of Allergy and Immunology, Faculty of Medicine, Saitama Medical University, Japan; b Department of Microbiology, Faculty of Medicine, Saitama Medical University, Japan; c Allergy Center, Saitama Medical University, Japan

**Keywords:** Herbal polyphenol, Tannic acid, Galloyl group, Dopamine receptor, Cytokine, Lipopolysaccharide, T cell receptor, T-helper, Inflammation, Inflammatory bowel disease

## Abstract

Tannic acid (TA) is an herbal polyphenol containing a galloyl group that has been prescribed to treat gastroenteritis, diarrhea, and irritable bowel syndrome. TA has anti-inflammatory, anti-cancer, and anti-viral properties; however, the molecular mechanisms of these potential therapeutic effects are still largely unknown. Here, we examined the ability of TA to induce anti-inflammatory responses. TA was found to be an agonist of the dopamine D2L receptor. TA reduced interferon (IFN)-γ and interleukin (IL)-1β secretion but upregulated tumor necrosis factor α and IL-10 secretion from lipopolysaccharide (LPS)-stimulated mouse splenocytes. TA also reduced IFN-γ secretion but enhanced IL-10 secretion from anti-cluster of differentiation (CD) 3/CD28 antibody-stimulated splenocytes. An immune subset study confirmed that TA regulated cytokine secretion by various types of immune cells in the context of stimulation with LPS or anti-CD3/CD28 antibodies. Administration of TA to mice with experimentally induced colitis strikingly suppressed weight loss, colon shrinkage, and IL-17 secretion from mesenteric lymph node lymphocytes in response to CD3/CD28 stimulation. These data suggest that TA suppresses inflammatory responses in colitis by regulating cytokine secretion by immune cells in the colon.

## Highlights

• Tannic acid is an agonist of the dopamine D2L receptor.
• Tannic acid suppresses IFN-γ secretion by LPS-stimulated splenocytes.
• Tannic acid modulates anti-CD3/CD28 antibody-stimulated cytokine levels in CD4^+^ T cells.
• Tannic acid ameliorates dextran sodium salt (DSS)-induced colitis in C57BL/6 mice.
• Tannic acid reduces production of IL-17 in DSS-induced colitis.

## Abbreviations:

TA: tannic acid
iNOS: inducible nitric oxide synthase
COX-2: cyclooxygenase-2
PP1: protein phosphatase 1
PP2A: protein phosphatase 2A
DA: dopamine;
D2LR: D2L receptor
DAR: DA receptor
D1-like-R: D1-like receptor
D2-like-R: D2-like receptor
Th: T-helper
IL: interleukin
Mϕ: macrophage
DSS: dextran sodium salt
LPS: lipopolysaccharide;
PBS(-): phosphate buffered saline without calcium/magnesium
HBSS: Hank’s Balanced Salt Solution
EC_80_: 80% effective concentration
RLU: relative light units
BM-DC: bone marrow-derived dendritic cell
GM-CSF: granulocyte-macrophage colony stimulating factor
CD: cluster of differentiation
BM: bone marrow
MAPK: mitogen-activated protein kinase
ERK1/2: extracellular signal-regulated kinase 1/2
JNK: c-jun N-terminal kinase
MLN: mesenteric lymph node
IFN: interferon
TNF: tumor necrosis factor
ANOVA: analysis of variance
ED: a fragment β-galactosidase enzyme donor
TCR: T cell receptor
IBD: inflammatory bowel disease
AD: Alzheimer’s disease
NK: natural killer
PD: Parkinson’s disease
SD: standard deviation

## 1 Introduction

Tannins are water-soluble herbal polyphenols (Erdelyi et al., 2005) and can be categorized into four major groups: gallotannins, which are also known as tannic acid (TA); ellagitannins; complexes of TA and ellagitannins; and condensed tannins. TA is hydrolyzable and is the most abundant tannin. The typical structure of TA is five polygalloyl esters binding radially to one glucose molecule through ester bonds.

TA has been prescribed to treat gastroenteritis (Michalek et al., 2016; Ruszczynski et al., 2014) and acute diarrhea (Lambelin, 2013). TA can also react with protein in the mucous membrane, causing it to precipitate and form a thin layer on top of the mucosa that protects it from inflammatory damage (de Jesus et al., 2012). In addition, TA directly suppresses inflammation (Feldman et al., 2001; Mota et al., 1985), cancer cell proliferation (Fong et al., 1972), and the proliferation of viruses in host cells (Uchiumi et al., 1996).

It has been suggested that the anti-inflammatory effects of TA are induced through the scavenging of radicals (antioxidant effect) (Hagerman et al., 1999) and inhibition of the expression of inflammatory mediators such as cytokines (Feldman et al., 2001), inducible nitric oxide synthase (iNOS), and cyclooxygenase-2 (COX-2) (Lee et al., 2003). Inhibition of the expression of the inflammatory mediators should be dependent on the inhibition of cell signaling. Indeed, it has been shown that TA inhibits protein phosphatase 1 (PP1) and 2A (PP2A) in epithelial cells (Erdelyi et al., 2005). However, the molecular mechanisms through which TA inhibits cell signaling are largely unclear.

Here, we show that TA is a dopamine (DA) D2L receptor (D2LR) agonist. The DA receptor (DAR) family consists of five cognate receptors, D1R, D2R, D3R, D4R, and D5R. D2R possesses two isoforms called D2SR (short isoform) and D2LR (long isoform). The five DARs belong to a superfamily of membrane proteins called the G-protein-coupled receptor family of class A seven-transmembrane domain receptors. These are sub-categorized as D1-like receptors (D1-like-Rs) (D1R and D5R) and D2-like receptors (D2-like-Rs) (D2R, D3R, and D4R), depending on their effect on the cytosolic cAMP level.

In neuronal cells, stimulation of D1-like-Rs triggers cAMP synthesis, which in turn activates protein kinase A. By contrast, stimulation of D2-like-Rs triggers cAMP degradation. In addition, stimulation of DARs increases intracellular calcium, which in turn activates calcium-binding proteins such as protein kinase C. D2R signaling induces activation of phosphoinositide 3-kinase, followed by activation of Akt and mammalian target of rapamycin (Girgis et al., 2008; Nair and Sealfon, 2003). On the other hand, the D2R-mediated signaling is desensitized and dampened by β-arrestin 2 recruitment (Amar et al., 2008).

DARs are expressed on various immune cells, and DA is also produced by these cells (Arreola et al., 2016; Nakano et al., 2009). It has been suggested that DA modulates the cytokine profile during inflammatory immune responses (Arreola et al., 2016) and facilitates T-helper (Th) 2 and Th17 differentiation (Nakano et al., 2009, 2011). DAR antagonists have anti-inflammatory effects (Kawano et al., 2015), and D1R antagonists in particular have been found to ameliorate various inflammatory autoimmune diseases (Hashimoto et al., 2009; Nakagome et al., 2011; Nakano et al., 2008, 2011; Okada et al., 2009). In a previous study, D1R-mediated signaling suppressed interleukin (IL)-1β processing and secretion in bone marrow-derived macrophages (Mϕs) (Yan et al., 2015). In addition, D2R knockout mice showed inflammatory responses in the central nervous system (Shao et al., 2013). These observations collectively indicate that modulation of the constitutive and/or temporal activities of DARs by DAR agonists and antagonists induces an anti-inflammatory response (Kenakin, 2001; Nicola et al., 2000; Prather, 2004). Hence, TA, acting via DARs, would also be expected to modulate the cytokine profile during inflammatory immune responses. In this study, we address the effect of TA on cytokine secretion during immune cell activation and its therapeutic activity in a dextran sodium salt (DSS)-induced mouse model of colitis (Kawano et al., 2015; Perse and Cerar, 2012; Yan et al., 2012). Our findings indicate that TA, which is found in tea, grains, and fruits, regulates immune responses by acting on DARs. It is therefore possible that TA could be used to suppress inflammation in the context of inflammatory disease.

## 2 Materials and methods

### 2.1 Reagents

TA was purchased from Wako (Osaka, Japan). Lipopolysaccharide (LPS) and L-741,626 were purchased from Sigma (St. Louis, MO). To make a stock solution, TA was dissolved in RPMI medium containing 10% fetal calf serum, 100 U/ml penicillin, 100 ​μg/ml streptomycin, 2 ​mM L-glutamine, and 50 ​μM 2-mercaptoethanol (R10 medium). Stock solutions of other chemicals were prepared by dissolving them in DMSO (Sigma).

### 2.2 D2LR agonist assay for TA

The cAMP modulation was determined using the HitHunter cAMP XS ​+ ​assay (Discoverx, Fremont, CA) (Eglen and Singh, 2003; Weber et al., 2004; Zhang and Xie, 2012). The cAMP Hunter CHO-K1 DRD2L (Long Isoform) Gi Cell Line (Discoverx) was seeded in a total volume of 20 ​μl in white-walled, 384-well microplates and incubated at 37 ​°C for the appropriate times prior to testing. For agonist determination, the cells were incubated with sample in the presence of forskolin at the EC_80_ (25 ​μM) to induce a response. After overnight incubation, medium was aspirated from the cells and replaced with 10 ​μl of HBSS and 10 ​mM HEPES. Stock solutions of the test compounds or DA were diluted to 4 ​× ​in assay buffer (Discoverx) immediately prior to use, and 5 ​μl of the 4 ​× ​dilution was added to the cells, followed by the addition of 5 ​μl of 4 ​× ​forskolin. The cells were then incubated at 37 ​°C for 30 ​min. Following incubation, an assay signal was generated by incubation with 5 ​μl of cAMP XS ​+ ​Ab reagent (Discoverx) and 20 ​μl of cAMP XS ​+ ​ED/CL lysis cocktail (Discoverx). After incubation for 1 ​h ​at room temperature, 20 ​μl of cAMP XS ​+ ​EA reagent (Discoverx) was added, and the cells were incubated for 2 ​h ​at room temperature. Microplates were read on a PerkinElmer Envision following chemiluminescent signal generation. Compound activity was analyzed using the CBIS data analysis suite (ChemInnovation). The percent agonist activity relative to the maximum effect of DA was calculated as follows: (mean RLU of control buffer - mean RLU of test sample)/(mean RLU of control buffer - mean RLU of the maximum response by DA) ​× ​100, where the control buffer was Opti-MEM (Thermo Fisher Scientific, Waltham, MA) supplemented with 100 U/ml penicillin, 100 ​μg/ml streptomycin, 2 ​mM L-glutamine, and 1% BSA.

### 2.3 Mice

C57BL/6 mice were obtained from Japan SLC (Shizuoka, Japan). Mice were housed in appropriate animal care facilities at Saitama Medical University, and handled according to international guidelines for experiments with animals. All experiments in the present study were approved by the Animal Research Committee of Saitama Medical University.

### 2.4 Preparation of splenocytes

Spleens were excised from C57BL/6 mice, placed in 10 ​ml of RPMI medium, and teased apart with tweezers to release splenocytes. The mixture was then centrifuged, and the supernatant was discarded. The cell pellet was resuspended in 250 ​μl of 0.75% (w/v) ammonium chloride and 17 ​mM Tris-HCl (pH 7.2) to rupture red blood cells and then mixed immediately with 10 ​ml of RPMI and centrifuged. After removing the supernatant, the cell pellet was washed three times with 10 ​ml of RPMI medium and resuspended in 1 ​ml of R10 medium. Splenocytes were then counted using a hemocytometer. The purity and viability of splenocytes was routinely >90% (Sup. Fig. 1 and Sup. Table 1, respectively).

### 2.5 Preparation of peritoneal Mϕs

Peritoneal Mϕs were isolated from C57BL/6 mice on day 4 post-intraperitoneal injection of 3 ​ml of 3% thioglycolate broth (Nissui Pharmaceutical, Tokyo, Japan). Briefly, 10 ​ml of RPMI was injected into the peritoneal cavity using a 21-gauge needle. The cavity was flushed three times with the same 10 ​ml of RPMI medium, which was collected at the end of the procedure. The medium was centrifuged for 5 ​min ​at 270×*g* at room temperature, and the cells were resuspended in 1 ​ml of R10 medium and counted. The cells were then transferred to 6-well polystyrene plates (3 ​× ​10^6^ ​cells/well) and incubated for 1 ​h ​at 37 ​°C in a CO_2_ incubator. Non-adherent cells were removed, and adherent cells (the peritoneal Mϕs) were scraped using a rubber policeman and counted. The purity and viability of peritoneal Mϕs were >90% and approximately 50%, respectively (Sup. Fig. 1 and Sup. Table 1, respectively).

### 2.6 Preparation of bone marrow-derived dendritic cells (BM-DCs)

Bone marrow-derived dendritic cells (BM-DCs) were prepared as described previously (Lutz et al., 1999). Briefly, the femurs and tibiae of C57BL/6 mice were removed and cleaned of surrounding muscle tissue by scraping with a knife. Then, both ends of the bones were cut with scissors and the bones were flushed with 1 ​ml of RPMI medium; the resulting marrow was collected in a 15 ​ml tube. After centrifuging at 270×*g* for 5 ​min ​at room temperature, the cell pellet was resuspended in 1 ​ml of R10 medium and counted. On day 0, the bone marrow leukocytes (2 ​× ​10^6^ ​cells in 10 ​ml of R10 medium containing 20 ​ng/ml granulocyte-macrophage colony stimulating factor (GM-CSF); R&D Systems, Minneapolis, MN) were seeded in a 100 ​mm dish. On day 3, another 10 ​ml of R10 medium containing 20 ​ng/ml GM-CSF was added. On days 6 and 8, half of the culture supernatant was collected and centrifuged. The resulting cell pellet was resuspended in 10 ​ml of fresh R10 medium containing 20 ​ng/ml GM-CSF and returned to the original dish. On day 10, the cells (now termed BM-DCs) were washed, resuspended in 1 ​ml of R10 medium, and counted. The purity and viability of BM-DCs was >90% (Sup. Fig. 1 and Sup. Table 1, respectively).

### 2.7 Isolation of cluster of differentiation (CD) 4^+^ T cells, CD8^+^ T cells, B cells, neutrophils, and monocytes

Mouse cluster of differentiation (CD) 4^+^ T cells, CD8^+^ T cells, B cells, monocytes, and neutrophils were isolated by positive selection from C57BL/6 splenocytes (CD4^+^ T cells, CD8^+^ T cells, and B cells) or from C57BL/6 bone marrow (BM) leukocytes (neutrophils) or negative selection from C57BL/6 BM leukocytes (monocytes) by magnetic-activated cell sorting (Miltenyi Biotec, Bergisch Gladbach, Germany) according to the manufacturer’s instructions. Each cell type was resuspended in 1 ​ml of R10 medium and counted. The purity and viability of CD4^+^ T cells, CD8^+^ T cells, B cells, and neutrophils was >90% (Sup. Fig. 1 and Sup. Table 1, respectively). The purity and viability of monocytes were >80% and 90%, respectively (Sup. Fig. 1 and Sup. Table 1, respectively).

### 2.8 Isolation of naïve CD4^+^ T cells

Mouse CD4^+^CD62L^+^ T cells were isolated by a combination of negative selection and positive selection from C57BL/6 splenocytes by magnetic-activated cell sorting (Miltenyi Biotec), according to the manufacturer’s instructions. The CD4^+^CD62L^+^ T cells were resuspended in 1 ​ml of R10 medium and counted. Since the percentage of CD4^+^CD44^−^ T cells was >90% of the isolated CD4^+^CD62L^+^ T cells, the purity and viability of naïve CD4^+^ T cells was >90% (Sup. Fig. 2 and Sup. Table 1, respectively).

### 2.9 Immune cell activation in the presence of TA

For CD3/CD28 stimulation, splenocytes (3 ​× ​10^5^), CD4^+^ T cells (3 ​× ​10^5^), or CD8^+^ T cells (3 ​× ​10^5^) in 50 ​μl of R10 medium were seeded in 96-well flat-bottom plates. Next, 50 ​μl of TA (4 or 40 ​μg/ml in R10 medium) or R10 medium alone was added for 1 ​h ​at 37 ​°C in a CO_2_ incubator. After incubation, 100 ​μl of R10 medium containing 2 ​μg/ml anti-mouse CD3 antibody (R&D Systems), and 1 ​μg/ml anti-mouse CD28 antibody (Biolegend, San Diego, CA) or PBS(-) (diluted 1:250) was added. The final concentration of TA was 1 or 10 ​μg/ml in 200 ​μl of R10 medium, and the final concentrations of anti-mouse CD3 antibody and anti-mouse CD28 antibody were 1 ​μg/ml and 0.5 ​μg/ml, respectively. The viability of CD3/CD28-stimulated immune cells treated with 1 or 10 ​μg/ml TA was no different from that of CD3/CD28-stimulated immune cells not treated with TA (Sup. Table 2). The cells were incubated for 24 ​h ​at 37 ​°C in a CO_2_ incubator, and then the culture supernatant was collected and centrifuged, and supernatants were collected for cytokine measurement by ELISA. For LPS stimulation, splenocytes (3 ​× ​10^5^), B cells (3 ​× ​10^5^), neutrophils (2 ​× ​10^5^), monocytes (5 ​× ​10^4^), peritoneal Mϕs (1 ​× ​10^5^), or BM-DCs (3 ​× ​10^5^) in 50 ​μl of R10 medium were seeded in 96-well flat-bottom plates. Next, 50 ​μl of TA (4 or 40 ​μg/ml in R10 medium) or R10 medium alone was added for 1 ​h ​at 37 ​°C/5% CO_2_. After incubation, 100 ​μl of R10 medium containing LPS (1 ​μg/ml) or DMSO (diluted 1:1000) was added. The final concentration of TA was 1 or 10 ​μg/ml in 200 ​μl of R10 medium, and the final concentration of LPS was 0.5 ​μg/ml. The viability of LPS-stimulated immune cells treated with 1 or 10 ​μg/ml TA was no different from that of LPS-stimulated immune cells not treated with TA (Sup. Table 3). The cells were incubated for 24 ​h ​at 37 ​°C in a CO_2_ incubator, and then the culture supernatant was collected and supernatants were collected for cytokine measurement by ELISA.

### 2.10 Phosphorylation of mitogen-activated protein kinases (MAPKs) in immune cells activated in the presence of TA

To examine whether TA affected the phosphorylation of signaling molecules in LPS-stimulated splenocytes, 50 ​μl of splenocytes (3 ​× ​10^5^ ​cells for analysis of extracellular signal-regulated kinase 1/2 (ERK1/2) and p38 mitogen-activated protein kinase (MAPK); 3 ​× ​10^6^ ​cells for analysis of c-jun N-terminal kinase (JNK)) were seeded in 96-well round-bottom plates. Next, 50 ​μl of TA (4 or 40 ​μg/ml in R10 medium) or R10 medium alone was added to the cells for 1 ​h. Then, 100 ​μl of R10 medium containing LPS (1 ​μg/ml), 2 ​μg/ml anti-mouse CD3 antibody (R&D systems), and 1 ​μg/ml anti-mouse CD28 antibody (Biolegend), DMSO (diluted 1:1000), or PBS(-) (diluted 1:250) was added for 15 ​min. The final concentration of TA was 1 or 10 ​μg/ml in 200 ​μl of R10 medium; cells were stimulated with a final concentration of 0.5 ​μg/ml LPS or 1 ​μg/ml anti-mouse CD3 antibody and 0.5 ​μg/ml anti-mouse CD28 antibody. After stimulation, the cells were centrifuged, and the pellet was resuspended in 200 ​μl of 1 ​× ​SDS-PAGE loading buffer to prepare cell lysates. The cell lysates were sonicated and boiled at 98 ​°C for 5 ​min, and 10% (v/v) of each sample was separated by SDS-PAGE on 10% gels, followed by Western blotting with antibodies against phosphorylated ERK1/2, JNK, or p38 MAPK (Cell signaling technology, Danvers, MA). As a control, expression of total ERK1/2, JNK, and p38 MAPK in the lysates was also analyzed (Cell signaling technology). Densitometric measurement was performed using Image J software (Schneider et al., 2012). Fold changes were calculated by dividing each density value by that of the medium control.

### 2.11 Naïve CD4^+^ T cell differentiation in the presence of TA

For naïve CD4^+^ T cell differentiation, naïve CD4^+^ T cells (3 ​× ​10^5^) in 125 ​μl of R10 medium were seeded in 24-well flat-bottom plates. Next, 125 ​μl of TA (4, 8, 20, or 40 ​μg/ml in R10 medium) or R10 medium alone was added for 1 ​h ​at 37 ​°C in a CO_2_ incubator. After incubation, 250 ​μl of R10 medium containing 2 ​μg/ml of anti-mouse CD3 antibody (R&D Systems) and 1 ​μg/ml of anti-mouse CD28 antibody (Biolegend, San Diego, CA) without (for spontaneous Th1 skewing) or with (for Th17 skewing) mouse IL-6 (40 ​ng/ml) (Peprotech, Rocky Hill, NJ) and human TGF-β1 (4 ​ng/ml) (Peprotech) was added. The final concentration of TA was 1, 2, 5, or 10 ​μg/ml in 500 ​μl of R10 medium, and the final concentrations of anti-mouse CD3 antibody and anti-mouse CD28 antibody were 1 ​μg/ml and 0.5 ​μg/ml, respectively, with or without IL-6 (20 ​ng/ml) and TGF-β1 (2 ​ng/ml). The viability of CD3/CD28-stimulated immune cells treated with 1, 2, 5, or 10 ​μg/ml TA with or without IL-6 and TGF-β1 was the same as that of CD3/CD28-stimulated immune cells not treated with TA (Sup. Table 4). The cells were incubated for 7 days at 37 ​°C in a CO_2_ incubator, and then the culture supernatants were collected, centrifuged, and used for cytokine measurement by ELISA.

### 2.12 Immune cell activation in the presence of TA and a D2R antagonist

For D2R antagonist treatment, wells of a 96-well plate were seeded with 50 ​μl of splenocytes (3 ​× ​10^5^ ​cells) in R10 medium, and 20 ​μl of R10 medium containing L-741,626 (a D2R antagonist) or DMSO (diluted 1:1000) was added to the wells for 1 ​h ​at 37 ​°C. Next, 50 ​μl of TA (40 ​μg/ml) in R10 medium, or R10 medium alone, was added to the wells for another 1 ​h. Splenocytes were then activated with 80 ​μl of LPS (1.25 ​μg/ml) dissolved in R10 medium, anti-mouse CD3 (2.5 ​μg/ml) and anti-mouse CD28 (1.25 ​μg/ml) antibodies dissolved in R10 medium, or the appropriate negative controls (R10 medium containing DMSO diluted 1:800 for LPS or PBS(-) diluted 1:200 for anti-CD3/CD28 antibodies) for 24 ​h. The final concentration of D2R antagonist was 1, 2, 5, or 10 ​μM in 200 ​μl of R10 medium containing splenocytes (3 ​× ​10^5^ ​cells); cell preparations were supplemented with 10 ​μg/ml TA, 0.5 ​μg/ml LPS, or 1 ​μg/ml anti-CD3 antibody and 0.5 ​μg/ml anti-CD28 antibody. The culture supernatant was collected, centrifuged, and used for cytokine measurement by ELISA. The viability of the splenocytes at 24 ​h after DMSO, LPS, PBS(-), or anti-CD3/CD28 antibody treatment with or without TA in the presence or absence of the D2R antagonist is shown in Supplementary Table 5.

### 2.13 Induction of experimental colitis of mice with DSS

Induction of experimental colitis of mice was induced by DSS as described previously (Kawano et al., 2015; Yan et al., 2012). C57BL/6 mice were housed for 7 days in a conventional animal facility at the Saitama Medical University before being used in the study. The mice were provided with drinking water containing 0, 1, or, 5 ​mg/ml TA for 3 days before the induction of experimental colitis. To induce colitis, the mice were provided with drinking water containing 4% DSS with 0, 1, or 5 ​mg/ml TA for 4 days, and then drinking water with TA alone (0, 1, or 5 ​mg/ml TA) for another 3 days. Body weights were recorded during colitis induction. The mice were sacrificed on day 7, their colon lengths were measured, and their mesenteric lymph nodes (MLNs) were excised.

### 2.14 Histological analysis of mouse colons

Histological analysis of the colon was performed by excising the colon at day 7 after colitis induction. Paraffin-embedded tissue sections of the whole colon were stained with hematoxylin and eosin to assess colon injury and inflammation (New histo science laboratory, Tokyo, Japan). Tissue sections were observed using the light microscopy mode of an inverted fluorescence phase-contrast microscope (BZ-9000, Keyence, Osaka, Japan).

### 2.15 Preparation of lymphocytes from MLNs

To extract MLN lymphocytes, the MLNs were excised, placed into 10 ​ml of RPMI medium, and dissociated with tweezers to discharge the lymphocytes into the medium. The medium was passed through a cell strainer with a 40 ​nm mesh (Corning, Corning, NY) and then centrifuged. The supernatant was discarded, and the cell pellet was resuspended in 1 ​ml of R10 medium. MLN lymphocytes (1 ​× ​10^5^ ​cells) were then stimulated with 1 ​μg/ml anti-mouse CD3 antibody and 0.5 ​μg/ml anti-mouse CD28 antibody in 200 ​μl of R10 medium in 96-well flat-bottom plates. After 24 ​h, supernatants were collected, and interferon (IFN)-γ and IL-17 levels were analyzed by ELISA.

### 2.16 Cytokine ELISAs

Levels of IFN-γ (range, 15.6–2,000 ​pg/ml), tumor necrosis factor (TNF)-α (range, 15.6–2,000 ​pg/ml), IL-1β (range, 15.6–1,000 ​pg/ml), IL-4 (range, 15.6–1,000 ​pg/ml), IL-5 (range, 15.6–2,000 ​pg/ml), IL-6 (range, 15.6 to 1,000 ​pg/ml), or IL-17 (15.6 to 1,000 ​pg/ml) in cell supernatants were measured using specific ELISAs (DuoSet kits, R&D Systems), according to the manufacturer’s instructions. Any value under the lower limit of detection of 15.6 ​pg/ml was set to 0. No cytokine cross-reactivity was observed within the detection ranges of the kits. If necessary, samples were diluted appropriately so that the cytokine level fell within the detection range for each cytokine.

### 2.17 Statistical analysis

Differences between more than three groups were analyzed by one-way analysis of variance (ANOVA) with Tukey’s post-hoc tests. Calculations were performed using KaleidaGraph software (Synergy software, Reading, PA). A P-value of <0.05 was considered statistically significant.

## 3 Results

### 3.1 TA acts as an agonist of D2LR

**Fig. 1:**
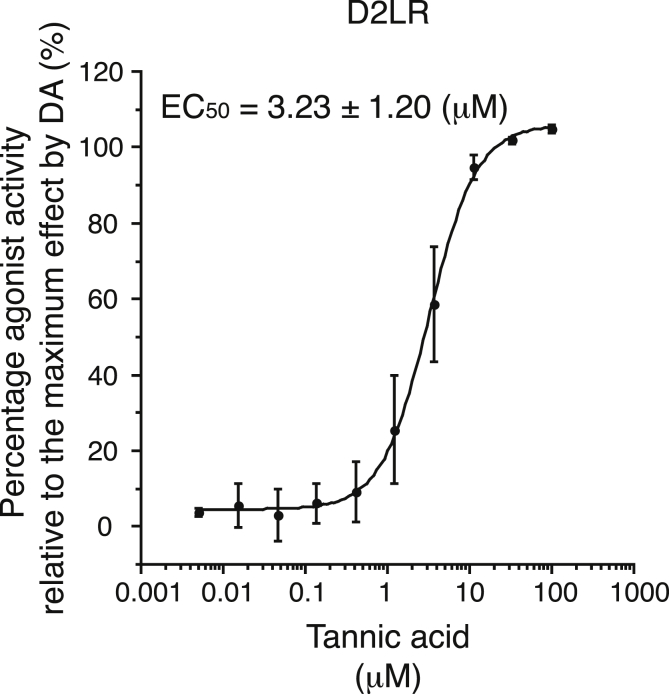
**Tannic acid (TA) acts as an agonist of the dopamine D2L receptor (D2LR).** Percentage activities (%) of D2LR are plotted in serial dilutions of tannic acid (TA). The experiments were performed four times and the EC_50_ value of TA for D2LR was calculated.

In our previous studies, a D1R antagonist, SCH2 3390, ameliorated various types of autoimmune inflammation (Hashimoto et al., 2009; Nakagome et al., 2011; Nakano et al., 2008, 2011; Okada et al., 2009). In agreement with these findings, we found that berberine, an herbal alkaloid that is prescribed for gastroenteritis and diarrhea, is a D1R and D2LR antagonist and ameliorated DSS-induced experimental colitis in mice (Kawano et al., 2015). We hypothesized that other herbal compounds used to treat gastroenteritis or diarrhea may act on DARs. During the screening, TA was found to be a D2LR agonist, and the EC_50_ of TA for D2LR agonism was calculated (Fig. 1). For this calculation, we used a cell line that exogenously overexpresses D2L and was engineered to detect increases in intracellular cAMP levels in response to agonism of the receptor (Eglen and Singh, 2003; Weber et al., 2004; Zhang and Xie, 2012). Since D2LR is coupled with endogenously expresses Gi and D2LR agonism induces cAMP degradation, forskolin at the EC_80_ was added prior to and during this experiment to induce cAMP synthesis. To determine the percent agonist activity on D2LR relative to the maximum effect of DA, TA (3-fold serial dilutions, starting at 100 ​μM ​TA) was added and incubated with the cell line to induce cAMP degradation. The cAMP level was determined using a competitive immunoassay. The remaining endogenous cAMP and exogenously added cAMP that was conjugated with a fragment β-galactosidase enzyme donor (ED) were competitively captured with a cAMP antibody. Then, the uncaptured ED-conjugated cAMP complexed to complement with the β-galactosidase acceptor, forming an active β-galactosidase. The active β-galactosidase activity was detected by chemiluminescence. Hence, a decrease in the intracellular cAMP level correlates with a decrease in the chemiluminescent signal. In parallel, the cell line was incubated with DA (3-fold serial dilutions, starting at 1 ​μM DA) and the maximum cAMP degradation by DA was determined. The EC_50_ of TA on D2LR was calculated as 3.23 ​± ​1.20 ​μM. This result suggests that TA acts as an agonist of D2LR.

### 3.2 TA modulates cytokine secretion by mouse splenocytes

**Fig. 2:**
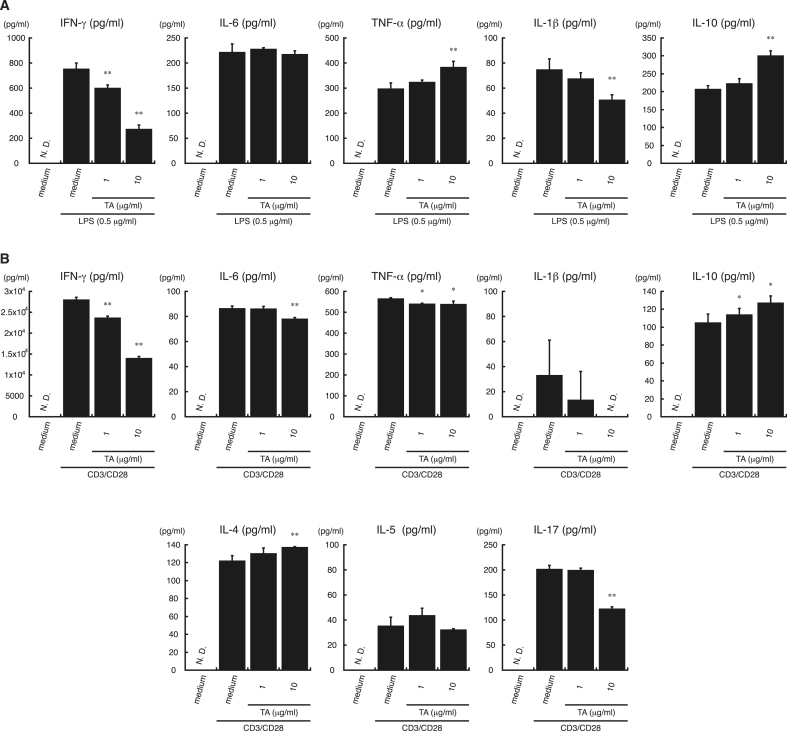
**TA modulates cytokine secretion by lipopolysaccharide (LPS)- or cluster of differentiation (CD) 3/CD28-stimulated splenocytes.***A*, Splenocytes from C57BL/6 mice were incubated with TA (1 or 10 ​μg/ml) or medium alone for 1 ​h, followed by stimulation with lipopolysaccharide (LPS) (0.5 ​μg/ml) or medium alone for 24 ​h. Next, supernatants were collected and interferon (IFN)-γ, interleukin (IL)-6, tumor necrosis factor (TNF)-α, IL-1β, and IL-10 levels were measured by ELISA. *B*, Splenocytes from C57BL/6 mice were incubated with TA (1 or 10 ​μg/ml) or medium alone for 1 ​h, followed by stimulation with anti-cluster of differentiation (CD) 3 antibody (1 ​μg/ml) and anti-CD28 antibody (0.5 ​μg/ml) (CD3/CD28) or medium alone for 24 ​h. Supernatants were collected, and IFN-γ, IL-6, TNF-α, IL-1β, IL-10, IL-4, IL-5, and IL-17 were measured by ELISA. All experiments were repeated at least three times. Data are expressed as the mean ​± ​standard deviation (SD) and were analyzed by one-way analysis of variance (ANOVA) with Tukey’s post-hoc tests. ∗P ​< ​0.05 and ∗∗P ​< ​0.01, compared with CD3/CD28-stimulated samples not treated with TA. N.D., not detected.

Since DA can regulate cytokine secretion during innate immune responses (Arreola et al., 2016), we next asked whether TA could regulate immune activity in a similar manner. Mouse splenocytes were treated with increasing concentrations of TA for 1 ​h and then stimulated with LPS for 24 ​h. After stimulation, levels of IFN-γ, IL-6, TNF-α, IL-1β, and IL-10 in the supernatant were analyzed by ELISA. As shown in Fig. 2A, secretion of IFN-γ and IL-1β was suppressed by the addition of TA in a dose-dependent manner, but secretion of TNF-α and IL-10 was enhanced by TA.

We next addressed whether TA also modulated cytokine secretion from splenocytes treated with immunostimulants other than LPS. For this purpose, splenocytes were treated with TA for 1 ​h and then stimulated with anti-CD3/CD28 antibodies for 24 ​h to activate T cells within the splenocytes. As shown in Fig. 2B, TA significantly suppressed IFN-γ and IL-17 secretion but enhanced IL-4 and IL-10 secretion in a dose-dependent manner. Treatment with TA alone (1 or 10 ​μg/ml) did not induce the secretion of any of the cytokines tested (data not shown). Since IFN-γ (Hsieh et al., 1993; Scharton and Scott, 1993), IL-4 (Bottomly, 1988; Min et al., 2004; Shinkai et al., 2002), and IL-17 (Kimura and Kishimoto, 2010; Kolls and Linden, 2004; Nakae et al., 2002) are characteristic cytokines produced by activated Th1, Th2, and Th17 ​cells, respectively, these suggested that TA would suppress Th1 and Th17 immune responses but enhance Th2 immune responses.

### 3.3 TA does not inhibit MAPK signaling in splenocytes

**Fig. 3:**
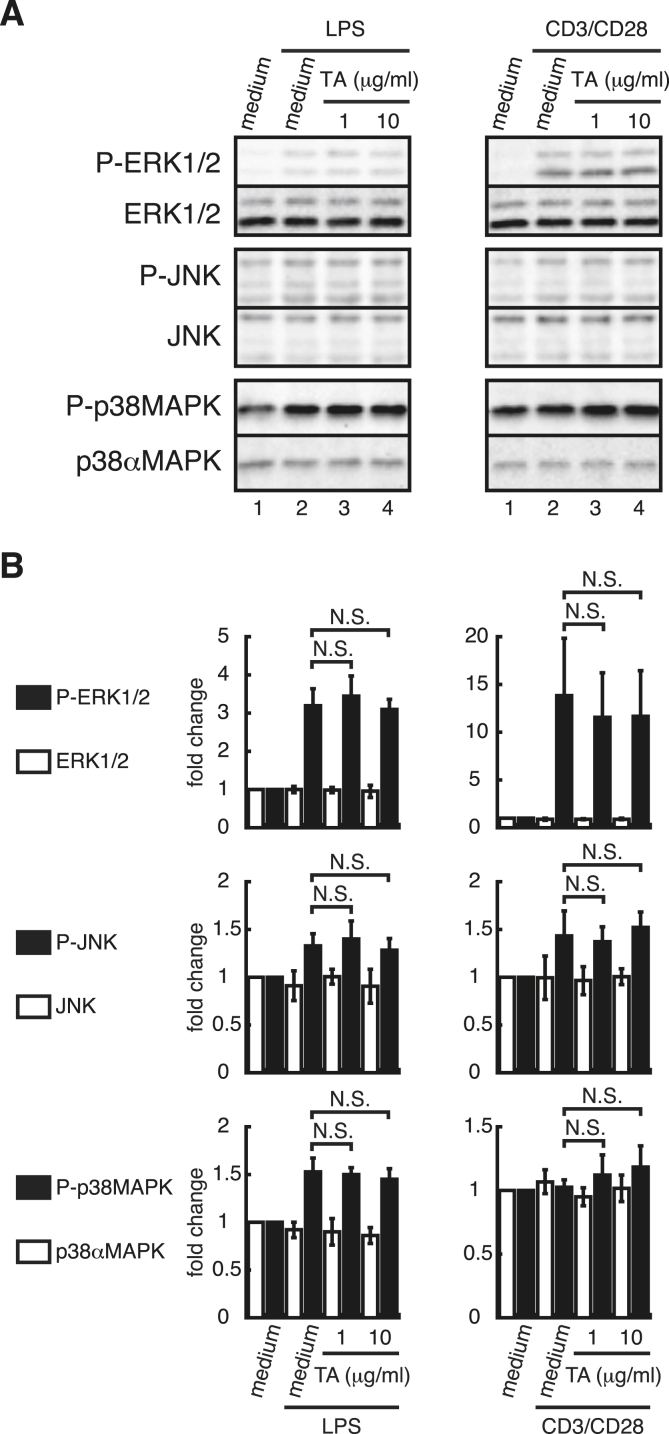
**TA did not affect mitogen-activated protein kinase (MAPK) signaling in LPS- or CD3/CD28-stimulated splenocytes.***A*, Western blots of phosphorylated and non-phosphorylated signaling molecules detected with specific antibodies. Splenocytes from C57BL/6 mice were incubated with TA (1 or 10 ​μg/ml) or medium for 1 ​h. Each sample was then stimulated with LPS (0.5 ​μg/ml) (*left*), anti-CD3 antibody (1 ​μg/ml), and anti-CD28 antibody (0.5 ​μg/ml) (CD3/CD28) (*right*), or medium alone, for 15 ​min and then spun down. The cell pellet was resuspended in 1 ​× ​SDS-PAGE loading dye, sonicated, boiled, and subjected to SDS-PAGE followed by Western blotting using anti-phosphorylated extracellular signal-regulated kinase 1/2 (ERK1/2), c-jun N-terminal kinase (JNK), and p38 mitogen-activated protein kinase (MAPK) antibodies. As a control, the expression of total ERK1/2, JNK, and p38 MAPK was also analyzed. Data from representative experiments are shown. All experiments were repeated at least three times. *B,* Densitometric analysis of the Western blot data in A for each MAPK. Analyses were performed using blots for each MAPK from the same gel. The fold change was determined by dividing the density value of each stimulated sample by that of the medium control for each MAPK. All experiments were repeated at least three times. Data are expressed as the mean ​± ​SD and were analyzed by one-way ANOVA with Tukey’s post-hoc tests. N.S., not significant.

In our previous study (Kawano et al., 2018), DA regulated cytokine secretion by LPS-stimulated splenocytes without suppressing MAPK signaling. This raised the possibility that TA also modulated cytokine secretion by LPS- or CD3/CD28-stimulated splenocytes without suppressing MAPK signaling. To address this, splenocytes were treated for 1 ​h with TA, followed by stimulation with LPS or anti-CD3/CD28 antibodies for 15 ​min. TA had no effect on the phosphorylation of ERK1/2, JNK, or p38 MAPK after stimulation with LPS (Fig. 3A, lanes 2–4, and Fig. 3B, left panels) or anti-CD3/CD28 antibodies (Fig. 3A, lanes 2–4, and Fig. 3B, right panels). Treatment with TA alone (1 or 10 ​μg/ml) did not induce the phosphorylation of ERK1/2, JNK, or p38 MAPK (data not shown). This suggests that TA does not alter LPS- or T cell receptor (TCR)-mediated MAPK signaling.

### 3.4 TA modulates cytokine secretion by immune cell subsets

Next, to determine which immune subsets contributed to TA-mediated cytokine secretion following stimulation with LPS or anti-CD3/CD28 antibodies, each immune subset was purified and exposed to TA for 1 ​h, followed by stimulation with LPS or anti-CD3/CD28 antibodies for 24 ​h. Cytokine production was measured by ELISA.

**Fig. 4:**
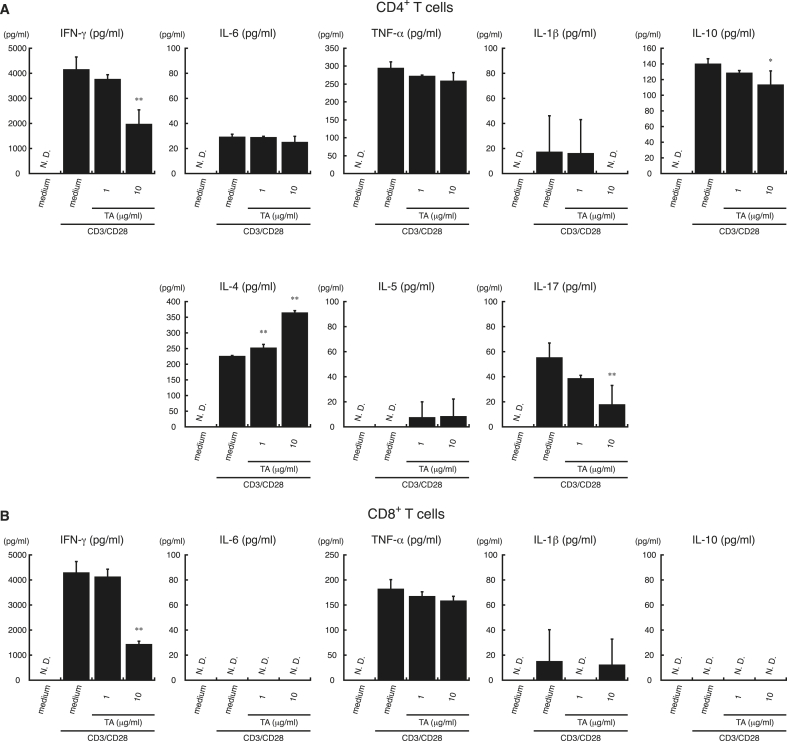
**TA modulates cytokine secretion by CD3/CD28-stimulated CD4**^**+**^**T cells.** CD4^+^ T cells (*A*) and CD8^+^ T (*B*) cells from C57BL/6 mice were incubated with TA (1 or 10 ​μg/ml) or medium alone for 1 ​h, followed by stimulation with anti-mouse CD3 antibody (1 ​μg/ml) and anti-mouse CD28 antibody (0.5 ​μg/ml) (CD3/CD28) or medium alone for 24 ​h. Supernatants were collected, and IFN-γ, IL-4, IL-5, IL-6, IL-17, TNF-α, IL-1β, and IL-10 levels were measured by ELISA. All experiments were repeated at least three times. Data are expressed as the mean ​± ​SD and were analyzed by one-way ANOVA with Tukey’s post-hoc tests. ∗P ​< ​0.05 and ∗∗P ​< ​0.01, compared with CD3/CD28-stimulated samples not treated with TA. N.D., not detected.

As shown in Fig. 4, TA suppressed the secretion of IFN-γ by CD4^+^ T cells and CD8^+^ T cells stimulated with anti-CD3/CD28 antibodies in a dose-dependent manner. TA also suppressed IL-17 secretion by CD3/CD28-stimulated CD4^+^ T cells in a dose-dependent manner, but enhanced IL-4 secretion. Since LPS does not induce cytokine production in T cells (Komai-Koma et al., 2004; Zanin-Zhorov et al., 2007), we did not analyze cytokine secretion by LPS-stimulated CD4^+^ or CD8^+^ T cells. These results suggest that TA can suppress IFN-γ secretion by TCR-stimulated CD4^+^ T cells and CD8^+^ T cells. Furthermore, suppression of IFN-γ and IL-17 secretion but upregulation of IL-4 secretion by TCR-stimulated CD4^+^ and CD8^+^ T cells indicates that TA suppresses Th1 and Th17 immune responses but enhances Th2 immune responses.

**Fig. 5:**
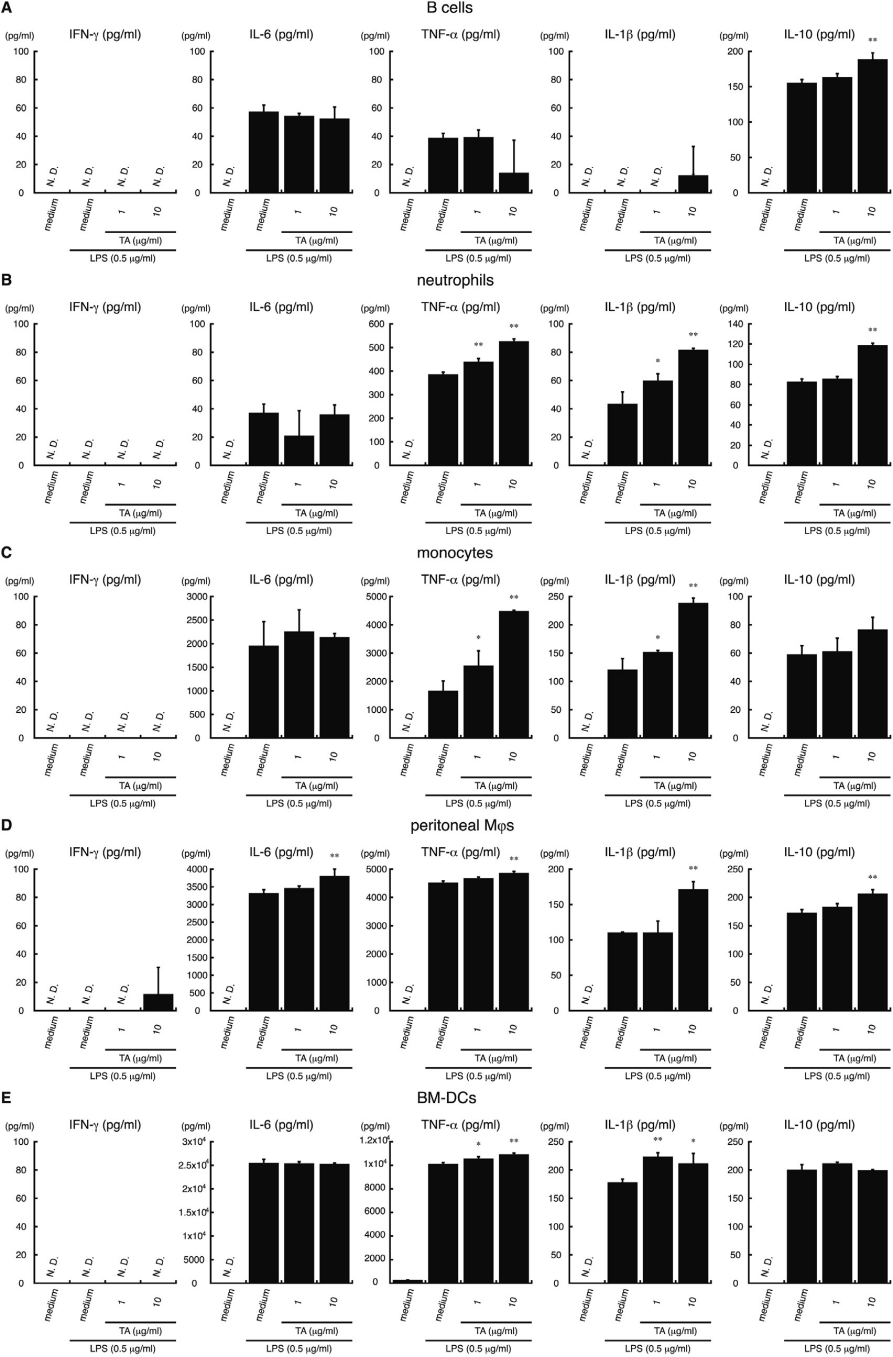
**Effect of TA on cytokine secretion by LPS-stimulated B cells, neutrophils, monocytes, peritoneal macrophages (Mϕs), and bone marrow-derived dendritic cells (BM-DCs).** B cells, neutrophils, monocytes, peritoneal macrophages (Mϕs), and bone marrow-derived dendritic cells (BM-DCs) from C57BL/6 mice were incubated with TA (1 or 10 ​μg/ml) or medium alone for 1 ​h, followed by stimulation with LPS (0.5 ​μg/ml) or medium for 24 ​h. IFN-γ, IL-6, TNF-α, IL-1β, IL-4, and IL-10 levels in supernatants from B cells (*A*), neutrophils (*B*), monocytes (*C*), peritoneal Mϕs (*D*), and BM-DCs (*E*) were measured by ELISA. All experiments were repeated at least three times. Data are expressed as the mean ​± ​SD and were analyzed by one-way ANOVA with Tukey’s post-hoc tests. ∗P ​< ​0.05 and ∗∗P ​< ​0.01, compared with LPS-stimulated samples without TA. N.D., not detected.

TA also significantly enhanced TNF-α and IL-1β secretion by LPS-stimulated neutrophils, monocytes, peritoneal Mϕs, and BM-DCs and IL-10 secretion by LPS-stimulated B cells, neutrophils, and peritoneal Mϕs (Fig. 5). In addition, TA enhanced the secretion of IL-6, which facilitates differentiation to a Th17 phenotype, by LPS-stimulated peritoneal Mϕs (Fig. 5) (Mosser and Edwards, 2008). Treatment with TA alone (1 or 10 ​μg/ml) did not induce cytokine secretion from any of the immune cell subsets tested (data not shown). These results suggest that TA-treated B cells, neutrophils, and peritoneal Mϕs were the source of the increased IL-10 in supernatants from LPS-stimulated splenocytes. TA also enhanced the secretion of TNF-α and IL-1β by LPS-stimulated neutrophils, monocytes, peritoneal Mϕs, and BM-DCs.

### 3.5 The effect of TA on the generation of Th1 and Th17 ​cells from naïve CD4^+^ T cells

**Fig. 6:**
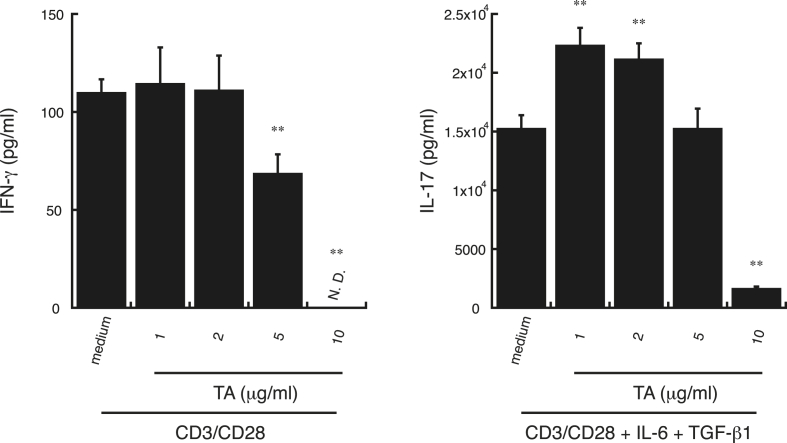
**Effect of TA on naïve CD4**^**+**^**T cell differentiation.** Naïve CD4^+^ T cells from C57BL/6 mice were incubated with TA (1, 2, or 5 ​μg/ml) or medium alone for 1 ​h, followed by stimulation with anti-mouse CD3 antibody (1 ​μg/ml) and anti-mouse CD28 antibody (0.5 ​μg/ml) (CD3/CD28) supplemented without (*left*) or with (*right*) mouse IL-6 (20 ​ng/ml) and human TGF-β1 (2 ​ng/ml) for 7 days. IFN-γ levels in supernatants from CD3/CD28-stimulated naïve CD4^+^ T cells (*left*) and IL-17 levels in supernatants from CD3/CD28-stimulated naïve CD4^+^T cells supplemented with IL-6 and TGF-β1 (*right*) were measured by ELISA. All experiments were repeated at least three times. Data are expressed as the mean ​± ​SD and were analyzed by one-way ANOVA with Tukey’s post-hoc tests. ∗∗P ​< ​0.01, compared with CD3/CD28-stimulated samples without TA. N.D., not detected.

It was hypothesized that TA might suppress the generation of Th1 cells by upregulating IL-10 secretion from activated B cells, neutrophils, and peritoneal Mϕs, resulting in a relatively high proportion of Th2 and Th17 ​cells compared with Th1 cells. To analyze the direct effect of TA on naïve CD4^+^T cell differentiation, CD3/CD28-stimulated naïve CD4^+^ T cells were incubated with TA for 7 days in the presence or absence of IL-6 and TGF-β1 to promote Th17 ​cell differentiation (Kimura and Kishimoto, 2010; Kolls and Linden, 2004; Nakae et al., 2002). Spontaneous secretion of IFN-γ, which is the characteristic cytokine produced by activated Th1 cells (Hsieh et al., 1993; Scharton and Scott, 1993), decreased in the presence of 5 and 10 ​μg/ml TA (Fig. 6, left panel), suggesting that Th1 generation was suppressed by TA at concentrations of 5 and 10 ​μg/ml. On the other hand, IL-17 secretion that was induced by the addition of IL-6 and TGF-β1 increased in the presence of 1 and 2 ​μg/ml TA, although IL-17 secretion was decreased at 10 ​μg/ml TA (Fig. 6, right panel), suggesting that Th17 differentiation was increased by TA in the 1–2 ​μg/ml range and was suppressed by TA at a concentration of 10 ​μg/ml. These results suggest that TA can directly suppress the generation of Th1 and Th17 ​cells from naïve CD4^+^ T cells by TA at a concentration of 10 ​μg/ml.

### 3.6 D2R antagonism partially reversed the modulation of cytokine secretion by TA

**Fig. 7:**
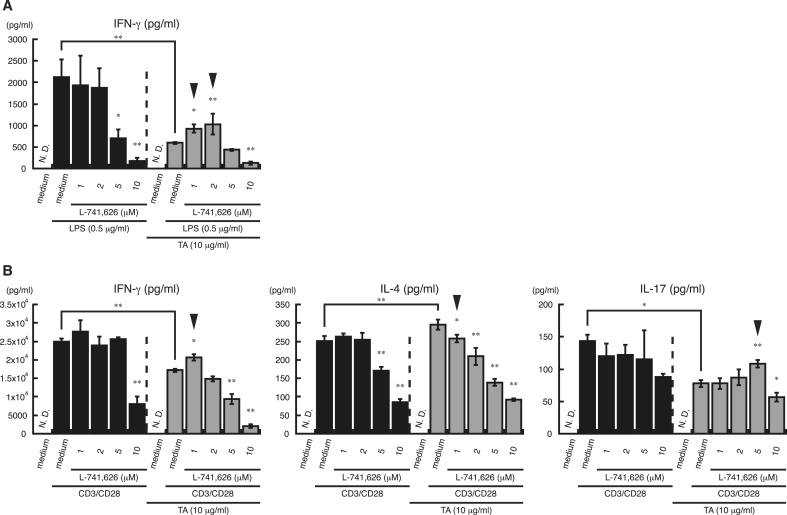
**Effect of a dopamine D2 receptor antagonist on cytokine secretion by LPS- or CD3/CD28-stimulated splenocytes.***A and B*, Splenocytes from C57BL/6 mice were incubated with D2R antagonist (L-741,626) or with medium containing DMSO (*control*) for 1 ​h, treated with TA (10 ​μg/ml) or medium alone, and then stimulated with LPS (0.5 ​μg/ml) or medium containing DMSO (*A*), or anti CD3/CD28 antibodies (CD3/CD28) or medium containing PBS(-) (*B*) for 24 ​h. Supernatants were then collected, and IFN-γ, IL-4, and IL-17 levels were measured by ELISA. All experiments were repeated at least three times. Data are expressed as the mean ​± ​SD. One-way ANOVA with Tukey’s post-hoc tests were performed for comparison of data. ∗P ​< ​0.05, ∗∗P ​< ​0.01, compared with LPS-stimulated samples containing DMSO (*A, left*) or LPS-stimulated samples containing DMSO and TA (10 ​μg/ml) (*A, right*). ∗P ​< ​0.05, ∗∗P ​< ​0.01, compared with CD3/CD28-stimulated samples containing PBS(-) (*B, left panels*) or CD3/CD28-stimulated samples containing PBS(-) and TA (10 ​μg/ml) (*B, right panels*). N.D., not detected.

Our data indicated that TA-mediated D2LR stimulation modulated cytokine secretion by splenocytes in the presence of LPS and anti-CD3/CD28 antibodies. It was therefore expected that a D2R antagonist would reverse the modulation of cytokine secretion by TA. To address this, splenocytes were incubated with the potent and selective D2R antagonist L-741,626 (Bowery et al., 1996), and then TA was added, followed by LPS (Fig. 7A) or anti-CD3/CD28 antibodies (Fig. 7B). As expected, the suppression of LPS- and anti-CD3/CD28-antibody-induced IFN-γ secretion by TA was partially reversed by the D2R antagonist at 1 and 2 ​μM (Fig. 7A, black arrowheads) and 1 ​μM (Fig. 7B, left panel, black arrowhead), respectively. In addition, the suppression of IL-17 secretion by TA was partially reversed by the D2R antagonist at 5 ​μM (Fig. 7B, right panel, black arrowhead). The upregulation of IL-4 secretion by TA was suppressed by the D2R antagonist at 1 ​μM (Fig. 7B, center panel, black arrowhead), and at a 1 ​μM concentration of the D2R antagonist, there was no significant difference in IL-4 secretion in the presence of both TA and the D2R antagonist compared with the D2R antagonist alone. As observed previously (Kawano et al., 2018), a high concentration (10 ​μM) of the D2R antagonist inhibited cytokine secretion by immune cells. These results indicate that TA modulates cytokine secretion by direct stimulation of D2LR.

### 3.7 TA ameliorates inflammation in a mouse model of colitis

TA suppressed IFN-γ secretion by CD4^+^ T cells stimulated with anti-CD3/CD28 antibodies, suggesting that TA can suppress Th1 and Th17 immune responses. This result suggests that TA may be able to ameliorate inflammatory bowel disease (IBD), including Crohn’s disease, which is dependent on Th1 and Th17 immune responses (Hill and Artis, 2010; Strober et al., 2002). Indeed, it has been reported that some foods containing TA may ameliorate colitis (Orsi et al., 2014; Otari et al., 2012; Ritchie et al., 2017), but the role of TA in the anti-inflammatory properties of these foods has not been clearly addressed.

**Fig. 8:**
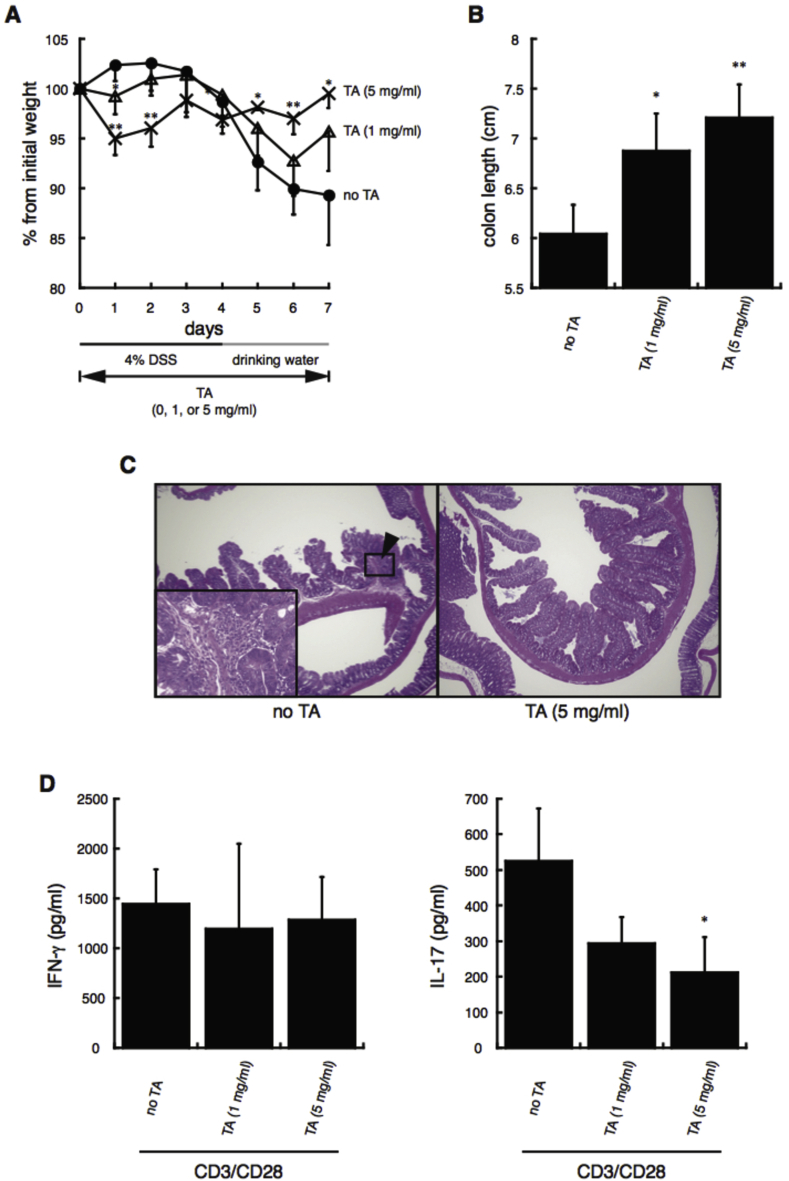
**Effects of TA on experimentally induced colitis in C57BL/6 mice.***A*, Body weights of mice were monitored during the 7 days of experimental colitis induction (4 days of 4% dextran sodium salt (DSS) water containing 0, 1, or 5 ​mg/ml TA, followed by 3 days of normal tap water containing 0, 1, or 5 ​mg/ml TA). The percent change from the initial weight (vertical axis) on each day (horizontal axis) was calculated by setting the weight at day 0–100%. All experiments were repeated at least three times. *B*, Mice were sacrificed after 7 days of colitis induction, and their colons were excised and measured. ∗P ​< ​0.05 and ∗∗P ​< ​0.01, compared with mice given 4% DSS water and normal drinking water without TA. All experiments were repeated at least three times. *C,* Paraffin-embedded colon sections were stained with hematoxylin and eosin for assessment of colon inflammation. Mice were administered drinking water containing 4% DSS with 0 or 5 ​mg/ml TA for 4 days, and then drinking water with 0 (*left*) or 5 ​mg/ml TA (*right*) for another 3 days. The black arrowhead indicates infiltrating lymphocytes. Insets in the *left* panel show the characteristic lymphocyte infiltration at a higher magnification. All experiments were repeated at least three times. Representative images are shown. *D*, IFN-γ and IL-17 secretion by CD3/CD28-stimulated mesenteric lymph node (MLN) lymphocytes obtained from mice with experimentally induced colitis given drinking water containing 4% DSS with 0, 1, or 5 ​mg/ml TA for 4 days, and then drinking water containing 0, 1, or 5 ​mg/ml TA for another 3 days. On day 7 after colitis induction, mice were sacrificed, MLN lymphocytes were harvested and stimulated with anti-CD3/CD28 antibodies, and secretion of IFN-γ (*left*) and IL-17 (*right*) was analyzed. All experiments were repeated at least three times. ∗P ​< ​0.05, compared with CD3/CD28-stimulated MLN lymphocytes from mice given 4% DSS water and normal drinking water without TA.

The effect of TA was therefore analyzed in a DSS-induced mouse model of colitis (Kawano et al., 2015; Perse and Cerar, 2012; Yan et al., 2012). To induce colitis, C57BL/6 mice were provided with drinking water containing 4% DSS with or without 1 or 5 ​mg/ml TA for 4 days, and then given drinking water with or without 1 or 5 ​mg/ml TA for an additional 3 days (Fig. 8). The percent change in the weight of the mice during the 7 days of experimental colitis induction is shown in Fig. 8A. Severe weight loss was observed in the mice given no TA on days 5–7 of colitis induction. By contrast, reduced weight loss was observed on days 5–7 in the mice given 1 ​mg/ml TA, and significant inhibition of the weight loss was observed in the mice given 5 ​mg/ml TA.

Colon shrinkage was also improved in mice given 1 or 5 ​mg/ml TA (Fig. 8B). TA therefore showed therapeutic activity in a mouse model of colitis.

To observe inflammation of the colon, a histological study was performed at day 7 after colitis induction. Intestinal damage and clusters of infiltrating lymphocytes were observed on the surface of the mucosal layer in colon sections from mice given no TA (Fig. 8C, left, black arrowheads and insets). However, in the mice given 5 ​mg/ml TA, the intestinal damage and infiltrating lymphocytes were not observed (Fig. 8C, right), indicating that TA inhibited DSS-induced inflammation of the colon.

In Fig. 4A, TA suppressed IFN-γ and IL-17 secretion by TCR-stimulated CD4^+^ T cells. We therefore hypothesized that TA suppressed colitis by inhibiting T cell-mediated inflammatory immune responses. To confirm this, the effect of TA on lymphocytes isolated from the mesenteric lymph nodes (MLN) of mice with DSS-induced colitis was examined. Lymphocytes from the MLN of mice at 7 days after colitis induction were collected and stimulated with anti-CD3/CD28 antibodies to activate CD4^+^ T cells. As shown in Fig. 8D, MLN lymphocytes from mice given no TA secreted IFN-γ and IL-17 following TCR stimulation. IL-4 secretion was not detected. On the other hand, IL-17 production (Fig. 8D, right) was significantly decreased in the MLN lymphocytes of mice given 5 ​mg/ml TA. However, no significant difference in IFN-γ secretion (Fig. 8D, left) was observed between the groups, indicating that TA specifically suppressed Th17 immune responses in mice with DSS-induced colitis. These findings suggest that TA can suppress weight loss and colon inflammation by inhibiting Th17-driven immune responses.

## 4 Discussion

In this study, we demonstrate that TA acts as an agonist of D2LR, one of the D2-like-Rs. TA stimulation of immune cells upregulates constitutive and/or temporal activities of D2LR and its downstream signaling molecules, including Gi and phosphodiesterase (Kenakin, 2001; Nicola et al., 2000; Prather, 2004), and modulates immune cell cytokine secretion in response to LPS or TCR ligation. Since the characteristic structure of TA is five galloyl residues binding to one glucose molecule through ester bonds, we hypothesize that other natural compounds harboring galloyl residues, such as gallic acid, epicatechin gallate, and epigallocatechin gallate, may also act as agonists of D2LR. This hypothesis is supported by the finding that a proanthocyanidin-rich fraction containing an epicatechin gallate unit and an epigallocatechin gallate unit was able to stimulate D2R (DalBo et al., 2006; Savitri Kumar et al., 2009). We also hypothesized that TA would have similar effects on cytokine secretion as DA. In agreement with this, TA suppressed IFN-γ and IL-1β secretion but enhanced IL-10 secretion by LPS-stimulated splenocytes, which was also observed in response to DA treatment (Kawano et al., 2018). Moreover, we showed that TA modulated the secretion of IFN-γ by LPS-stimulated splenocytes and the secretion of IFN-γ, IL-4, and IL-17 by CD3/CD28-stimulated splenocytes; this modulation was partially reversed by the addition of a D2R antagonist, indicating that TA mainly acts on D2LR. However, as seen with other D2R agonists such as B-HT 920, which is also an α2 adrenoceptor agonist; 5-HT_3_ antagonists (Maina and Mathews, 2010; Nishio et al., 1996; Ricci and Taira, 1999); and rotigotine, which is also a D3R agonist (Scheller et al., 2009), TA may have agonist or antagonist effects on G protein-coupled receptors other than D2LR. In addition, in the previous study, DA did not suppress MAPK signaling in splenocytes stimulated with LPS but did suppress IFN-γ secretion (Kawano et al., 2018). As expected, TA also did not suppress MAPK signaling in LPS-stimulated splenocytes. It has been suggested that TA inhibits the enzymatic activity of poly(ADP-ribose) glycohydrolase (Aoki et al., 1993; Tsai et al., 1992) and may inhibit AP-1 transcriptional activity by inhibiting PP1 and PP2A (Erdelyi et al., 2005). These inhibitory effects of TA may contribute to its net effect on cytokine secretion by immune cells when TA is endocytosed and transferred into the cytosol of immune cells. There is a possibility that such cytosol-transferred TA may stimulate D2LR-mediated signaling molecules such as Gi and phosphodiesterases or inhibit adenyl cyclase activity, and that this could be responsible for the decrease in cAMP observed when cells were incubated with TA to detect D2LR agonist activity. The observation that TA-mediated modulation of cytokine secretion was partially reversed by the addition of the D2R antagonist suggests that TA mainly acts as a direct agonist of D2LR rather than by activating D2LR downstream signaling molecules. However, more precise studies are needed to elucidate the specific molecular mechanisms through which TA affects cytokine secretion.

TA also reduced mortality rates in patients with burn injuries (Hupkens et al., 1995) and has been shown to alleviate diarrhea (Lambelin, 2013; Michalek et al., 2016; Ruszczynski et al., 2014). In particular, albumin tannate is frequently prescribed for the treatment of diarrhea in Japan. Some foods containing TA have been shown to improve diabetes (Banihani et al., 2013), arthritis (Shukla et al., 2008), and even Alzheimer’s disease (AD) (Braidy et al., 2017; Fernando et al., 2017). These reports suggested that TA may have anti-inflammatory properties. However, TA also increased production of neutrophil chemoattractants, iNOS, and COX-2 from Mϕs (Rapizzi et al., 2004; Rohrbach et al., 1989), which are consistent with a proinflammatory role for TA.

In this study, TA suppressed IFN-γ and IL-17 secretion but upregulated IL-4 secretion from TCR-stimulated CD4^+^ T cells. TA also suppressed IFN-γ secretion from CD8^+^ T cells. IFN-γ production from TCR-stimulated CD4^+^ T cells was strongly suppressed by TA, but IL-17 secretion was only weakly suppressed by TA treatment, indicating that TA suppresses Th1 immune responses more strongly than Th17 responses. TA also suppressed IFN-γ production by LPS-stimulated natural killer (NK) cells (data not shown). By contrast, TA enhanced IL-4 secretion from TCR-stimulated CD4^+^ T cells. These data suggest that TA suppresses activated Th1 and Th17 responses but enhances Th2 immune responses. On the other hand, we noticed that IL-17 secretion by TCR-stimulated naïve CD4^+^ T cells in the presence of IL-6 and TGF-β1 was induced by TA in the 1–2 ​μg/ml range. One of the reasons of such different responses in IL-17 secretion between TCR-stimulated naïve CD4^+^ T cells and TCR-stimulated CD4^+^ T cells might be due to the differing TCR-coupled signaling pathways (Ahmadzadeh et al., 1999; Berard and Tough, 2002; Farber, 2009).

In addition, TA significantly enhanced TNF-α and IL-1β secretion by LPS-stimulated neutrophils, monocytes, peritoneal Mϕs, and BM-DCs. This is consistent with previous studies in which TA acted as a proinflammatory stimulus in macrophages (Rapizzi et al., 2004; Rohrbach et al., 1989), and with studies showing that TA enemas promoted extensive local inflammation in the colon and cecum, resulting in mucosal ulceration, acute hemorrhagic hepatic necrosis, and hemorrhagic cystitis (Dukes, 1975; Singleton, 1981). TA also significantly upregulated IL-10 secretion by LPS-treated B cells, neutrophils, and peritoneal Mϕs. TA-mediated upregulation of IL-10 might be an additional mechanism through which TA suppresses Th1 immune responses.

Overall, these data suggest that in the presence of TA, CD4^+^ T cells, CD8^+^ T cells, NK cells, B cells, and peritoneal Mϕs would inhibit Th1 and Th17 immune responses and promote Th2 immune responses. Suppression of Th1 and Th17 immune responses by TA is expected to result in reduced migration and activation of Mϕs and neutrophils at the site of inflammation, which may explain the therapeutic effect of TA in experimental colitis. Based on these results, we would hypothesize that the local inflammation, mucosal ulceration, hemorrhagic hepatic necrosis, and hemorrhagic cystitis (Dukes, 1975; Singleton, 1981) observed in response to TA enemas in previous studies could have resulted from TA activation of Mϕs and neutrophils in the absence of a polarized T cell response. Since TA significantly suppressed IFN-γ and IL-17 secretion by TCR-stimulated splenocytes, TA should suppress IFN-γ- and IL-17-mediated inflammatory responses. It was hypothesized that a D2R antagonist would inhibit the therapeutic effect of TA on DSS-induced colitis and might even exacerbate the colitis due to its ability to reverse TA-mediated modulation of cytokine secretion by LPS- and CD3/CD28-stimulated splenocytes (Fig. 7). However, when 1 or 10 ​μM D2R antagonist (L-741, 626) was added with 5 ​mg/ml TA in the drinking water prior to and during colitis induction, the D2R antagonist did not inhibit the therapeutic effects of TA, including inhibition of weight loss and colon shrinkage (data not shown). The failure of the D2R antagonist to inhibit the effects of TA on colitis induction may be related to the narrow effective range of the D2R antagonist on TA-mediated modulation of cytokine secretion by immune cells *in vitro*; it may be difficult to achieve an effective concentration of D2R antagonist *in vivo*. Another possibility is that the D2R antagonist failed to negate the therapeutic effects of TA and exacerbate DSS-induced colitis because it did not fully reverse the agonistic effects of TA on D2LR.

In our previous study, DA suppressed IFN-γ secretion by LPS-stimulated NK cells but enhanced IL-10 secretion from LPS-stimulated B cells, neutrophils, monocytes, peritoneal Mϕs, and BM-DCs. DA enhanced IL-6 secretion by LPS-stimulated peritoneal Mϕs but did not affect the secretion of TNF-α and IL-1β (Kawano et al., 2018). DA did not affect the secretion of IL-6, TNF-α, or IL-1β from LPS-stimulated BM-DCs (Kawano et al., 2018). Compared with DA, TA suppressed IFN-γ secretion by LPS-stimulated splenocytes but enhanced IL-10 secretion from LPS-stimulated splenocytes, B cells, neutrophils, and peritoneal Mϕs. TA also enhanced IL-6 secretion by LPS-stimulated peritoneal Mϕs. Therefore, the effects of DA and TA on cytokine secretion by immune cells are similar. These observations support our hypothesis that TA uses D2LR to modulate cytokine secretion by immune cells. However, TA, but not DA, enhanced TNF-α and IL-1β secretion by LPS-stimulated neutrophils, monocytes, peritoneal Mϕs, and BM-DCs. We hypothesize that when TA, which has a relatively high molecular weight of 1701.2 Daltons, endocytosed within the cells, TA might trigger inflammasome formation, resulting in IL-1β secretion (Shirasuna et al., 2019). However, the molecular mechanism through which TA enhances TNF-α secretion is unknown. The other difference between the effects of TA and DA was the relatively weak upregulation of IL-10 secretion by TA. One of the reasons for this might be that TA preferentially induces the secretion of TNF-α and IL-1β by monocytes, peritoneal Mϕs, and BM-DCs. TA, unlike DA, has been shown to inhibit poly(ADP-ribose) glycohydrolase (Aoki et al., 1993; Tsai et al., 1992) and PP1 and PP2A (Erdelyi et al., 2005). These inhibitory effects may contribute to the overall production of IL-10 by cells activated in the presence of TA. Interestingly, IL-10 upregulation was observed in TA-treated splenocytes stimulated with anti-CD3/CD28 antibodies, while TA treatment did not induce upregulation of IL-10 secretion from TCR-stimulated purified CD4^+^ T cells or CD8^+^ T cells. This may be because TCR-stimulated CD4^+^ T cells subsequently stimulate other immune cells such as B cells to secrete IL-10.

Administration of TA-containing extracts from foods such as fruits and vegetables has been shown to ameliorate IBD (Orsi et al., 2014; Yoshioka et al., 2008). In agreement with this finding, our data indicate that TA ameliorated colitis in a mouse model. TA reduced IL-17 secretion by MLN lymphocytes stimulated with anti-CD3/CD28 antibodies, indicating that TA suppresses Th17 immune responses in the colon, ameliorating the inflammation. In Fig. 4A, TA suppressed both IFN-γ and IL-17 secretion by CD4^+^ T cells stimulated with anti-CD3/CD28 antibodies, suggesting that TA can inhibit both Th1 and Th17 immune responses. It is possible that TA did not suppress IFN-γ secretion by MLN lymphocytes because it precipitates on top of the colon and does not penetrate into the colon tissue (de Jesus et al., 2012), resulting in the suppression of Th17 responses since Th17 preferentially accumulates on the colon membrane rather than Th1 in experimental colitis in mice. TA has been shown to partly precipitate and accumulate at the surface of the colon (de Jesus et al., 2012), which may increase its local concentration and enhance its efficacy, resulting in improvement of experimental colitis.

## 5 Conclusion

Taken together, these results indicate that TA may be effective in the treatment of Th1- and Th17-driven inflammatory diseases, including inflammatory autoimmune diseases such as nephritis, psoriasis, periodontal disease, encephalomyelitis, rheumatoid arthritis, neutrophilic airway inflammation, diabetes mellitus, and IBD. Moreover, there is some evidence that TA-containing foods may improve the symptoms of AD (Braidy et al., 2017; Fernando et al., 2017) and that DAR agonists may inhibit the progression of Parkinson’s disease (PD) (LeWitt, 2015; Rascol, 1999; Unti et al., 2015). TA may improve AD and PD by activating DARs in the central nervous system, similar to its effects on the enteric nervous system of the colon (Caputi and Giron, 2018; Sinclair et al., 2018; Zhu et al., 2018).

## Author contributions

M.K., K.S., and R.T. performed the experiments. M. K., K.S., R.T., and S.M. conceived and designed the experiments. M.K., M.M., and S.M. wrote the manuscript. All authors discussed the results and commented on the manuscript.

## Declaration of competing interest

We confirm that this manuscript has not been published elsewhere, nor is it under consideration by another journal and that, if accepted, it will not be published elsewhere in the same form, in English or in any other language, including electronically without the written consent of the copyright-holder. All financial supports including third party for the work were described at the Acknowledgments section in the manuscript. Sho Matsushita is an employee of iMmno, Inc. The authors have no other conflict interest of this work.
