# Supplementary material for: Tannic acid acts as an agonist of the dopamine D2L receptor, regulates immune responses, and ameliorates experimentally induced colitis in mice
Source: Brain Behav Immun Health. 2020 Apr 30;5:100071. doi: 10.1016/j.bbih.2020.100071 (PMC8474654; doi:10.1016/j.bbih.2020.100071)
Supplement: Multimedia component 1 [file mmc1.docx]

**Sup. Fig. 1. Percentages of each immune subset among splenocytes, bone marrow (BM) leukocytes, and isolated immune cell populations.** *First row*: Splenocytes or isolated CD4^+^ T cells were stained with a FITC-conjugated anti-mouse CD4 antibody (Ab) (BD Biosciences) (x-axis) and a PE-conjugated anti-mouse CD3 Ab (BioLegend) (y-axis). *Second row*: Splenocytes or isolated CD8^+^ T cells were stained with a FITC-conjugated anti-mouse CD8a Ab (BD Biosciences) (x-axis) and a PE-conjugated anti-mouse CD3 Ab (BioLegend) (y-axis). *Third row*: Splenocytes or isolated B cells were stained with a FITC-conjugated anti-mouse CD19 Ab (BioLegend) (x-axis), and cell numbers were counted (y-axis). *Fourth row*: Splenocytes, bone marrow (BM) leukocytes, or isolated neutrophils were stained with a FITC-conjugated anti-mouse CD11b Ab (BioLegend) (x-axis) and a PE-conjugated anti-mouse Gr-1 Ab (BioLegend) (y-axis). *Fifth row*: Splenocytes, BM leukocytes, and isolated monocytes were stained with a FITC-conjugated anti-mouse Ly-6C Ab (BioLegend) (x-axis) and a PE-conjugated anti-mouse CD115 Ab (BioLegend) (y-axis). *Sixth row*: Splenocytes and peritoneal macrophages (Mφs) were stained with a FITC-conjugated anti-mouse F4/80 Ab (BioLegend) (x-axis), and cell numbers were counted (y-axis). *Seventh row*: Splenocytes, BM leukocytes, and bone marrow dendritic cells (BM-DCs) were stained with a FITC-conjugated anti-mouse CD11c Ab (BioLegend) (x-axis), and cell numbers were counted (y-axis). The number in each panel represents the percentage of each immune subset within the total cell population.

**Sup. Fig. 2. Percentages of naïve CD4^+^ T cells.** *First row*: Splenocytes or isolated CD4^+^CD62L^+^ T cells were stained with a FITC-conjugated anti-mouse CD4 Ab (BioLegend) (x-axis) and a PE-conjugated anti-mouse CD62L Ab (BioLegend) (y-axis). *Second row*: Splenocytes or isolated CD4^+^CD62L^+^ T cells were stained with a FITC-conjugated anti-mouse CD4 Ab (BioLegend) (x-axis) and a PE-conjugated anti-mouse CD44 Ab (BioLegend) (y-axis). The number in each panel represents the percentage of each immune subset within the total cell population. **Supplementary Table 1.** **Viability of purified immune cells**

Cell type Viability

Splenocytes 100 ± 0

CD4^+^ T cells 100 ± 0

CD8^+^ T cells 100 ± 0

B cells 100 ± 0

Neutrophils 100 ± 0

Monocytes 100 ± 0

Peritoneal Mφs 52.2 ± 7.31

BM-DCs 100 ± 0

Naïve CD4^+^ T cells 100 ± 0

Cells were mixed with Trypan blue. Viability was calculated as the number of unstained cells/(stained cells + unstained cells) × 100. Each measurement was performed three times. The percentage listed in the table represents the average ± one standard deviation.

Mφs, macrophages.

BM-DCs, bone marrow-derived dendritic cells. **Supplementary Table 2. Viability of immune cells at 24 h post-treatment with anti-CD3/CD28 antibodies (CD3/CD28) and tannic acid (TA)**

Viability

Cell type Medium CD3/CD28 CD3CD/28 + 1 μg/ml TA CD3/CD28 + 10 μg/ml TA

Splenocytes 89.7 ± 2.33 93.2 ± 4.69 94.6 ± 1.85 92.2 ± 3.28

CD4^+^ T cells 90.2 ± 4.21 92.0 ± 2.56 94.6 ± 2.93 92.6 ± 3.84

CD8^+^ T cells 93.1 ± 1.36 96.2 ± 2.09 96.0 ± 0.910 94.4 ± 2.61

Cells were stained with Trypan blue at 24 h after stimulation with anti-CD3/CD28 antibodies. Viability was calculated as the number of unstained cells/(stained cells + unstained cells) × 100. Each measurement was performed three times. The percentage listed in the table represents the average ± one standard deviation.

TA, tannic acid. **Supplementary Table 3. Viability of immune cells at 24 h post-treatment with LPS and tannic acid (TA)**

Viability

Cell type Medium LPS LPS + 1 μg/ml TA LPS + 10 μg/ml TA

Splenocytes 87.9 ± 2.71 98.8 ± 1.32 98.9 ± 0.746 98.7 ± 0.792

B cells 85.6 ± 6.53 96.0 ± 2.45 95.7 ± 0.919 96.9 ± 1.52

Neutrophils 71.2 ± 8.91 86.3 ± 4.61 83.4 ± 5.34 78.0 ± 7.84

Monocytes 73.0 ± 4.97 93.4 ± 4.19 95.3 ± 3.28 86.8 ± 4.76

Peritoneal Mφs 89.4 ± 5.03 89.4 ± 2.39 89.9 ± 4.60 90.2 ± 0.839

BM-DCs 90.0 ± 4.02 98.5 ± 0.485 98.6 ± 1.50 99.0 ± 0.883

Cells were stained with Trypan blue at 24 h post-LPS stimulation. Viability was calculated as the number of unstained cells/(stained cells + unstained cells) × 100. Each measurement was performed three times. The percentage listed in the table represents the average ± one standard deviation.

LPS, lipopolysaccharide.

TA, tannic acid.

Mφs, macrophages.

BM-DCs, bone marrow-derived dendritic cells. **Supplementary Table 4. Viability of naïve CD4^+^ T cells at 7 days post-treatment with tannic acid (TA) and anti-CD3/CD28 antibodies**

Cell type

Naïve CD4^+^ T cells with anti-CD3/CD28 antibodies

TA concentration Viability

0 μg/ml TA 57.2 ± 9.54

0 μg/ml TA 57.2 ± 9.54

1 μg/ml TA 53.5 ± 10.1

2 μg/ml TA 61.1 ± 11.6

5 μg/ml TA 63.4 ± 13.8

10 μg/ml TA 66.0 ± 9.47

Cell type

Naïve CD4^+^ T cells with anti-CD3/CD28 antibodies in the presence of IL-6 + TGF-β1

TA concentration Viability

0 μg/ml TA 61.4 ± 3.67

1 μg/ml TA 62.0 ± 8.06

2 μg/ml TA 55.5 ± 3.40

5 μg/ml TA 52.5 ± 12.2

10 μg/ml TA 57.1 ± 10.4

Cells were stained with Trypan blue at 24 h after stimulation with LPS or anti-CD3/CD28 antibodies. Viability was calculated as the number of unstained cells/(stained cells + unstained cells) × 100. Each measurement was performed three times. The percentage listed in the table represents the average ± one standard deviation.

LPS, lipopolysaccharide.

TA, tannic acid.

**Supplementary Table 5. Viability of splenocytes at 24 h post-treatment with 10 μg/ml tannic acid (TA) and LPS or anti-CD3/CD28 antibodies**

Viability

D2 antagonist (L-741,626) (μM) medium 10 μg/ml TA

0 μM with DMSO control 86.5 ± 9.81 91.6 ± 1.96

0 μM with LPS 95.5 ± 2.77 96.8 ± 0.880

1 μM with LPS 97.8 ± 2.82 93.1 ± 6.81

2 μM with LPS 95.9 ± 1.34 97.1 ± 3.38

5 μM with LPS 95.1 ± 3.85 96.3 ± 4.07

10 μM with LPS 92.0 ± 3.70 93.0 ± 3.71

0 μM with PBS(-) control 86.9 ± 7.30 85.7 ± 7.11

0 μM with anti-CD3/CD28 antibodies 95.1 ± 0.719 89.9 ± 4.87

1 μM with anti-CD3CD28 antibodies 90.9 ± 9.19 87.5 ± 9.85

2 μM with anti-CD3/CD28 antibodies 93.9 ± 7.35 88.7 ± 5.70

5 μM with anti-CD3/CD28 antibodies 95.4 ± 5.56 92.9 ± 8.25

10 μM with anti-CD3/CD28 antibodies 85.5 ± 4.80 89.5 ± 5.27

Cells were stained with Trypan blue at 24 h after stimulation with LPS or anti-CD3/CD28 antibodies. Viability was calculated as the number of unstained cells/(stained cells + unstained cells) × 100. Each measurement was performed three times. The percentage listed in the table represents the average ± one standard deviation.

LPS, lipopolysaccharide.

TA, tannic acid.
