# Supplementary figures and images for: Tannic acid acts as an agonist of the dopamine D2L receptor, regulates immune responses, and ameliorates experimentally induced colitis in mice
Source: Brain Behav Immun Health. 2020 Apr 30;5:100071. doi: 10.1016/j.bbih.2020.100071 (PMC8474654; doi:10.1016/j.bbih.2020.100071)

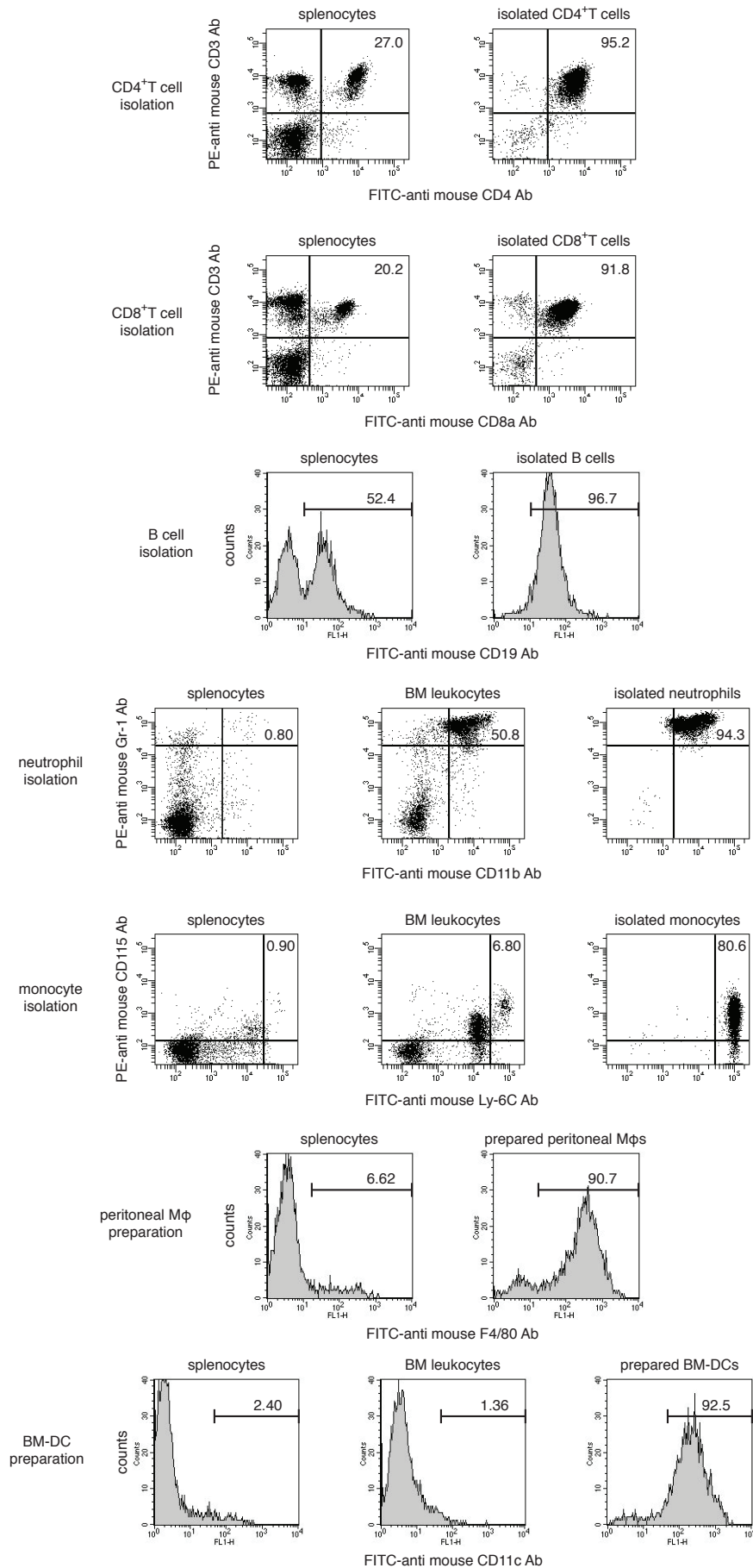

**Sup. Fig. 1**

Supplement: Multimedia component 2 [file mmc2.pdf]

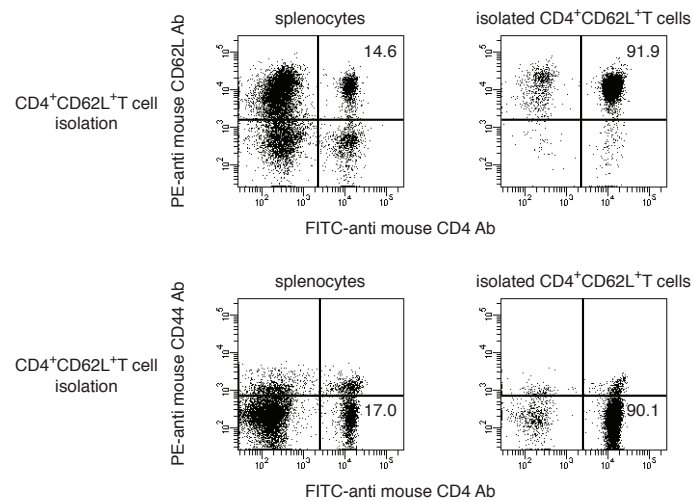

**Sup. Fig. 2**

Supplement: Multimedia component 3 [file mmc3.pdf]
